# Assessing oral health-related quality of life among older people in home-based care - survey results of the InSEMaP study in Germany

**DOI:** 10.1186/s12903-024-04500-6

**Published:** 2024-06-26

**Authors:** Alena Koenig, Sarah Porzelt, Anja Behrens-Potratz, Peter Stratmeyer, Stefanie Schellhammer, Petra Schmage, Claudia Konnopka, Martin Scherer, Alexander Konnopka, Thomas Zimmermann

**Affiliations:** 1 Department of Nursing and Management, Cooperative Process Management in Social and Health Care RTC (KoPM-Zentrum), Faculty of Business and Social Sciences, Hamburg University of Applied Sciences, Alexanderstraße 1, 20099 Hamburg, Germany; 2 Department of General Practice and Primary Care, Centre for Psychosocial Medicine, University Medical Centre Hamburg- Eppendorf, Hamburg, Germany Martinistraße 52, 20251; 3 Department of Periodontics, Preventive and Restorative Dentistry, Centre for Dental and Oral Medicine, University Medical Centre Hamburg-Eppendorf, Hamburg, Germany Martinistraße 52, 20251; 4 Department of Health Economics and Health Services Research, Centre for Psychosocial Medicine, University Medical Centre Hamburg-Eppendorf, Hamburg, Germany Martinistraße 52, 20251; 5 Department of Health Care Research and Innovation, Deutsche Angestellten Krankenkasse - Gesundheit (DAK-Gesundheit), Nagelsweg 27, 20097 Hamburg, Germany

**Keywords:** Oral Health Care, Oral Health Impact Profile, Home-based Care, Older people, Utilisation of Dental Care, Social Support, Oral health-related quality of life

## Abstract

**Background:**

Older people receiving home-based care (HBC) often face barriers to access preventive oral health care (OHC) and dental treatments. Leading to deterioration of their oral healthcare. It is further deteriorated by factors such as increasing burden of systemic diseases, medicinal side effects, limited mobility, financial constraints and lack of professional OHC at home. Older people also struggle to maintain necessary daily oral hygiene, leading to malnutrition, weight loss, and a risk of a further health degradation. This cross-sectional survey aimed to investigate the oral health-related quality of life (OHRQoL) and their associated factors in HBC recipients.

**Methods:**

5,280 older people (≥ 60 years) living in Hamburg, who were in need of care and insured with statutory health insurance DAK-Gesundheit received the questionnaire, which included the German version of the Oral Health Impact Profile (OHIP G-14) and, the EQ-5D health-related quality of life (HRQoL) measure as well as further questions regarding the extent of informal social support, subjective oral health status, oral health behaviour, subjective cognitive status, and socio-demographic variables.

**Results:**

The participants (*n* = 1,622) had a median age of 83.2 years, with 72.0% of the sample being female. Nearly two thirds of the sample reported that their independence or abilities were significantly impaired (care level 2). Regarding oral health impacts, 40.0% of the participants reported experiencing at least one of the fourteen possible prevalent impacts of the OHIP-G14 fairly often or very often. A multivariate regression model on the severity of oral health impacts revealed, that a better HRQoL, a positive perception of one’s own dental status, fewer visits to dental practices, and no need for support in OHC were associated with better OHRQoL. Conversely, respondents with a negative perception of their oral health status, more frequent visits to a dental practice, a need for support in OHC, and subjective memory impairment showed poorer OHRQoL.

**Conclusions:**

The results highlight the risk for poor oral health among older people in HBC. We conclude that there is an urgent need to prioritise oral health, especially as poor oral health can further compromise the systemic wellbeing of these already care dependent population.

**Supplementary Information:**

The online version contains supplementary material available at 10.1186/s12903-024-04500-6.

## Background

The global population of individuals aged 60 and over is projected to nearly double from 12.0% in 2015 to 22.0% in 2050 [1]. This demographic shift, both globally and in Germany, highlights the importance of focusing on oral health and its associated care. Oral health is integral to healthy ageing, as it interacts with many systemic diseases [2, 3].

However, a recent review by Poudel et al. [4] identified a dearth of information and research on effective oral health care (OHC) programs for geriatric care. Allen et al. [5] highlighted, a gap between research findings and their implementation into policy, emphasising the need for context-relevant research, particularly for the vulnerable group of frail older people.

Especially older people requiring home-based care (HBC) face challenges in accessing preventive OHC and dental treatments. Factors such as increasing burden of systemic diseases [6], side effects of medication intake (e.g. xerostomia) [7], restricted mobility [8], incomplete health coverage for oral diseases [9] and a lack of suitable facilities or transportation [10] contribute to deteriorating oral health in old age. This can lead to malnutrition, weight loss, and an increased risk of further general health deterioration, cardiovascular diseases, diabetes mellitus and cognitive impairment [3–5]. As these problems accumulate, older people find it increasingly difficult to maintain adequate daily oral hygiene [8]. Inadequate oral hygiene can lead to the accumulation of a pathogenic biofilm in the mouth within days [11]. Oral biofilm causes periodontal disease, but also has far-reaching systemic effects and is associated with numerous diseases [12].

Studies in Germany have shown that the rate of dental treatment decreases with an increased level of care and age [13, 14]. The 5th Oral Health Study in Germany found that older people in need of care received significantly less dental care than older people who do not require personal care [15]. Contrary to international findings [16], in Germany, the oral health of older people in HBC is worse than that of nursing home residents. This is primarily due to the fact that the dental care providers can visit in the inpatient settings through special contracts between long-term care facilities and dental practice [17]. Despite the growing body of knowledge on OHC for older people, a review of studies on oral health-related quality of life (OHRQoL) of older people in HBC revealed that these topics have not been extensively explored in research [18]. The concept of OHRQoL examines the impact of oral health on physical and psychosocial health [19], making it suitable for assessing the subjective oral health of older people [20–23].

The observational study “Interaction of Systemic Morbidity and Oral Health in Ambulatory Patients in Need of Home Care (InSEMaP)” was conducted to acknowledge the necessity for research on the oral health of older people in HBC settings. It aims to enhance understanding and provide insights for policymakers. This study investigates the perspectives of older people in HBC and those involved in the process of care. In addition to analysing claims data, a clinical examination was conducted by a dentist who visited older people in need of HBC at home [24]. The study is funded by the Innovation Fund of the Statutory Health Insurance Funds in Germany (Funding reference number: 01VSF20031). As part of the InSEMaP-project, a survey was conducted using a dental patient-reported outcome measure (dPROM) to assess the OHRQoL of older people in HBC.

### Aims

Thus, the aim of this survey was to investigate the OHRQoL and their associated factors in older people in need of HBC.

## Methods

### Design and setting

This study was a cross-sectional survey conducted among community dwelling older people, who were members of the DAK-Gesundheit statutory health insurance. The participants, whether they had an institutional caregiver or not, met the following inclusion criteria: they were ≥ 60 years old on December 31, 2021, required HBC according to the German Social Law Codebook XI, § 15 [25] since December 31, 2020, and lived in Hamburg. All insured older people who met the inclusion criteria were invited to participate in the survey between January and April 2022. In collaboration with DAK-Gesundheit, 5,280 older people meeting inclusion criteria were identified in its records. They were sent a postal mail which included a personalised invitation letter, a study information sheet, the questionnaire, and a prepaid return envelope. The questionnaire, which consisted of six sections (see supplementary material), did not include sender’s information to ensure that the responses were anonymised from the beginning. Cognitive pretesting was conducted with six subjects who met the inclusion criteria and were related to or associated with the research team. This pretesting led to a revised version of the questionnaire which was more suitable for the target population with more space between questions, a larger font and better accuracy.

All responses received by June 30, 2022, were analysed.

### Outcomes and measures

#### Primary outcome

The primary outcome was OHRQoL, which was measured using the German version of the 14-item Oral Health Impact Profile (OHIP-G14) [26]. The assessment encompassed questions related to functional limitations, physical pain, physical disability, psychological discomfort, psychological disability, social disability, and personal handicap. Each item was evaluated on a five-point Likert-scale, ranging from 0 to 4 (0 = never, 1 = hardly ever, 2 = occasionally, 3 = fairly often to 4 = very often) [27].

#### Further outcomes

The questionnaire also encompassed several sociodemographic characteristics, including sex (male or female or diverse), age and level of care. The latter is precisely in German Social Law Codebook XI, § 15 [25], and is officially determined by the Medical Service of the statutory health insurance. The need for care is graded into five levels (level 1: minor impairment of independence or abilities, level 2: considerable impairment of independence or abilities, level 3: severe impairment of independence or abilities, level 4: most severe impairment of independence or abilities, level 5: most severe impairment of independence and/or abilities with special care requirements).

Furthermore, education and vocational training were classified according to the international CASMIN classification [28] as either low (general elementary education or basic vocational training), medium (intermediate training or general maturity certificate), or high (lower or higher tertiary education).

Additional items included whether participants were living alone (yes vs. no), had a preferred dentist of their own choice (yes vs. no), or had a preferred general practitioner (GP) (yes vs. no). The utilisation of dental service since the onset of HBC was also assessed as: unchanged, more frequently, less frequently, or not at all.

Participants were asked to rate their subjective dental health status according to German Social Law, Codebook V, § 22a [29, 30]. The rating consisted of four items (condition of teeth; condition of mucous membranes/tongue/gums; condition of dentures; dental care), each rated as either very good, rather good (positive assessment), rather bad, or very bad (negative assessment).

A composite score was formed from these four items and then dichotomised: positive or negative assessments were summed up, composing a range of values − 4 to + 4. New groups were formed from this, representing a rather positive, a rather negative, or an undifferentiated (score = 0) overall assessment of the condition of one’s own subjective dental health status.

We inquired participants about their dental visits in the last 12 months with reference to the study titled “Current Health in Germany“ including the corresponding frequency of visits during this period [31]. Additionally, we assessed subjective oral care behaviour by examining the frequency of teeth brushing (not at all, irregular, once daily, twice daily, more than twice daily). Furthermore, participants were queried regarding their need for general support from caregivers and specific support related to oral and dental care (yes vs. no). In the latter case, participants could specify the individuals or services providing actual support.

To evaluate Health-Related Quality of Life (HRQoL), we employed the European Quality of Life 5 Dimensions measurement (EQ5D) [32] several domains such as: mobility, self-care, usual activities, pain/discomfort, and anxiety/depression. Responses were categorised as “no problems”, “slight problems”, “moderate problems”, “severe problems” to “extreme problems”. These responses were then transformed into an index score, adjusted for the German population [33]. Higher index values corresponded to better HRQoL.

As a self-assessment of cognitive condition, we employed a two-item screening test for subjective memory impairment (SMI) [34]. Participants were asked the following questions: “Do you feel that your memory has deteriorated?” (yes vs. no), and “If yes, are you concerned?” (yes vs. no). Only if participants answered “yes“ to both questions, their response were considered indicative of SMI – a recognised risk factor for the development of dementia [35].

Additionally, we inquired whether participants received assistance in completing the questionnaire (yes vs. no).

### Data Analysis

For the primary outcome OHIP-G14, three variables were computed, each representing distinct characteristics of OHRQoL [36]:

1. OHIP-G14 prevalence: the proportion of respondents who answered “fairly often” or “very often” to at least one of the OHIP-G14 items, expressed as a percentage.
2. OHIP-G14 extent: the sum of items (range 0–14) that were responded to “fairly often” or “very often”, expressed as mean and 95%-confidence intervals (CI).
3. OHIP-G14 severity: total summary score of all responses in OHIP-G14 (range 0–56), expressed as mean and 95%-CI.

Higher values in the extent and prevalence scores corresponded to more pronounced impairment of an individual’s oral health. These scores were calculated only for participants who provided responses to a minimum of 12 OHIP-items, in accordance with Slade et al. [36].

Additionally, categorical variables were computed as absolute and relative frequencies and percentages, while continuous variables were summarised as mean values along with their corresponding 95%-CI. To assess group differences, we employed specific statistical tests. For categorical variables related to OHIP-G14 prevalence impacts, we used the chi^2^ distribution test. For continuous variables specifically, dependent samples, we applied the t-test. Furthermore, we constructed a linear regression model using the OHIP-G14 severity score to identify factors associated with the severity of oral health impacts. A significance level of *p* < 0.05 was set for the alpha error to accept the hypotheses of potential group differences in bivariate analyses of OHIP-G14 prevalence or associations in multivariate analyses of OHIP-G14 severity. In the multivariate regression model, β-coefficients were computed, representing the slope of the specific association, where a one-unit change in the independent variable corresponds to a change of β in the dependent outcome, OHIP-G14 severity.

Data analyses were carried out using statistical analysis software Stata by StataCorp.

### Missing data

We conducted a thorough examination of missing data within the dataset considering three potential mechanisms for missingness: MCAR (Missing completely at random), MAR (Missing at Random), and MNAR (Missing Not At Random). Although, assuming MCAR is not very plausible for the dataset involving older people in HBC but there were numerous causes – both known and unknown – that could account for MAR or MNAR [37, 38]. It is reasonable to anticipate that older people in HBC had reasons for unknown origin including sudden medical situations, returning to daily routines after receiving the questionnaire, forgetting to complete it, or feeling fatigued during the process, – all of which fall under MNAR. On the other hand, MAR could be assumed when participants cancelled their response to the primary outcome OHIP-G14 before reaching the critical cutoff of 12 items. This cancellation might be influenced by variance in other (observed) variables, such as sex, age, or any other item in the questionnaire.

One approach to cross-validate results involved comparing a subset of the population with the entire population: in our study, we specifically compared two groups: respondents who completed the entire questionnaire (complete cases – CC, including partial responses) and respondents with valid questionnaires (encompassing all observed cases, - AOC). We then conducted separate analyses for summary statistics related to the primary outcome – prevalence, extent, severity of oral health impacts –and to assess missingness. Regression was performed as follows: logistic regression for examining prevalence and linear regression (twice) investigating extent and severity. These analyses were restricted to the sample of complete cases (CC). Subsequently, we compared the results from the CC-analyses with those obtained from the AOC-analysis.

### Reporting standard

To ensure that the survey is sufficiently reliable, reproducible and transparent, we used the reporting standard Checklist for Reporting of Survey Studies (CROSS statement) by Sharma et al. [39].

## Results

### Sample characteristics

The study included all questionnaires returned by June 30, 2022, with valid responses. Out of the 5,280 individuals invited, 1,622 (30.7%) responded, representing the AOC group. The Complete Cases (CC) group, comprising 1,371 respondents, included those who answered at least 12 items of the primary endpoint OHIP-G14 and indicated a care level between 1 and 5.

A summary table for both groups is provided (see Table 1). Of all possible values, 92.6% were recorded in the database, with 7.4% were missing.

**Table 1:** Summary statistic for all observed cases (AOC) and complete cases (CC)

AOC - all observed cases (*n* = 1,622)	Sum	Sum		CC - complete cases (*n* = 1,371)	Sum	Sum
	N	%			N	%
**Sex**						
Female	1,116	72.0		Female	994	72.0
Male	435	28.0		Male	386	28.0
Sum	1,551	100.0		Sum	1,380	100.0
**Level of care**						
1	15	1.0		1	12	0.9
2	984	63.7		2	882	64.3
3	409	26.5		3	370	27.0
4	104	6.7		4	81	5.9
5	32	2.1		5	26	1.9
Sum	1,544	100.0		Sum	1,371	100.0
**Education**						
Low	176	11.5		Low	158	11.6
Medium	1,153	75.5		Medium	1,026	75.3
High	198	13.0		High	178	13.1
Sum	1,527	100.0		Sum	1,362	100.0
**Living alone**						
No	729	49.0		No	656	49.3
Yes	758	51.0		Yes	675	50.7
Sum	1,487	100.0		Sum	1,331	100.0
**Have a preferred dentist**						
No	291	18.5		No	245	17.5
Yes	1,284	81.5		Yes	1,153	82.5
Sum	1,575	100.0		Sum	1,398	100.0
**Have a preferred GP**						
Other	132	8.6		Other	114	8.3
GP	1,397	91.4		GP	1,252	91.7
Sum	1,529	100.0		Sum	1,366	100.0
**Utilisation of dental care since HBC is needed**						
Unchanged	748	50.0		Unchanged	688	51.3
More frequently	112	7.5		More frequently	101	7.5
Less frequently / not at all	635	42.5		Less frequently / not at all	551	41.1
Sum	1,495	100.0		Sum	1,340	100.0
**Subjective dental status: mainly positive**						
No	462	29.3		No	399	28.5
Yes	1,113	70.7		Yes	999	71.5
Sum	1,575	100.0		Sum	1,398	100.0
**Subjective dental status: mainly negative**						
No	1,295	82.2		No	1,154	82.5
Yes	280	17.8		Yes	244	17.5
Sum	1,575	100.0		Sum	1,398	100.0
**Visited dentist in the past 12 months**						
No	295	21.3		No	259	20.8
Yes	1,093	78.7		Yes	989	79.2
Sum	1,388	100.0		Sum	1,248	100.0
**Frequency brushing teeth**						
Never	15	1.0		Never	14	1.0
Irregular	76	5.0		Irregular	60	4.5
Once daily	306	20.3		Once daily	268	19.9
Twice daily	924	61.3		Twice daily	841	62.6
More than twice daily	186	12.3		More than twice daily	161	12.0
Sum	1,507	100.0		Sum	1,344	100.0
**Need support for oral care**						
No	1,133	73.7		No	1,019	74.4
Yes	405	26.3		Yes	351	25.6
Sum	1,538	100.0		Sum	1,370	100.0
**At least one person for support of oral care**						
No support	648	48.2		No support	594	49.3
At least one person	697	51.8		At least one person	611	50.7
Sum	1,345	100.0		Sum	1,205	100.0
**Subjective memory impairment**						
No	811	51.5		No	723	51.7
Yes	764	48.5		Yes	675	48.3
Sum	1,575	100.0		Sum	1,398	100.0
**Support to fill in questionnaire**						
No	845	53.7		No	729	52.1
Yes	730	46.3		Yes	669	47.9
Sum	1,575	100.0		Sum	1,398	100.0
	**N**	**Mean**	**95%-CI**	**N**	**Mean**	**95%-CI**
**Age**						
	1,588	83.2	82.8–83.6		82.9	82.5–83.4
**Number of dental visits in the past 12 months**						
	1,412	2.0	1.8–2.1	1,248	2.0	1.9–2.1
**HRQoL Eq. 5D**						
	1,485	0.39	0.37–0.40	1,313	0.39	0.37–0.41

Level of care 1 to 5 (1 = minor impairment of independence or abilities, 5 = most severe impairment of independence and/or abilities with special care requirements); OHIP-G14: Oral Health Impact Profile German Version 14 items; GP: General Practitioner; HBC: home-based care; HRQoL EQ5D: Health-Related Quality of Life EuroQoL 5 Dimensions; CI: confidence interval

Nearly half of the participants in the AOC group received assistance in completing the questionnaire. The characteristics of both groups are presented in Table 1. Almost three quarters of the AOC-participants were female. Three quarters reported a medium level of education, and the mean age was 83.2 years. More than half of the respondents had care level 2, followed by care level 3 and 4. Half of the participants in the AOC group reported subjective memory impairment. In 91.4% of cases, the general practitioner is the first medical person to be consulted in the event of health problems (see Table 1, summary column).

If necessary, half of the sample received support with daily oral and dental care. Among the participants, four out of five had a preferred dentist for regular visits, and more than three quarters had visited a dentist in the last 12 months. Notably, over 40.0% reported visiting their dentist less frequently or not at all since becoming care dependent. On average, participants had 2.0 dental visits in the last 12 months.

In Table 2, we observed that 40.0% of the participants reported experiencing one or more oral health-related prevalent problems, as indicated by OHIP-G14 item(s) rated ‘fairly often’ or ‘very often’. When considering OHIP-G14 extent, 12.0% of the participants reported four and more impairments. The overall mean OHIP-G14 extent score was 1.5.

**Table 2:** Quantity of Oral Health-Related Problems and OHIP-G14 Extent

Quantity of Oral Health-Related Problems	*N*	%	Cumulative %
0	946	60.06	60.06
1–4	441	27,99	88.06
5–9	146	9.27	97.33
10–14	42	2.66	100.00
OHIP-G14 Extent			
	**N**	**Mean**	**[95%-CI]**
OHIP-G14 Extent all	1,575	1.5	[1.4,1.6]
OHIP-G14 Extent at least one problem	629	3.8	[3.6,4,0]

OHIP-G14: Oral Health Impact Profile German Version 14 items; CI: confidence interval

**Fig. 1:**
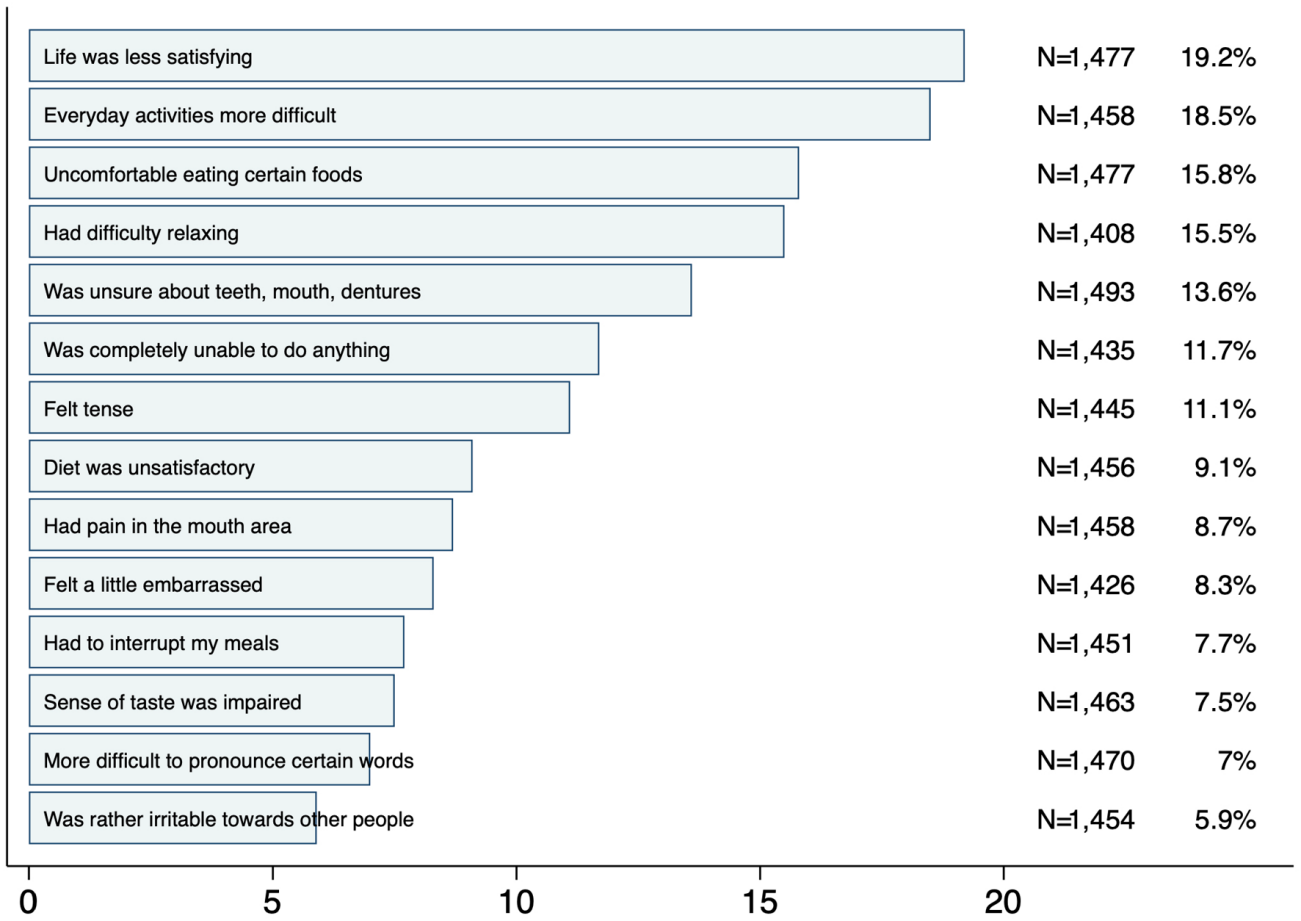
Ranking of oral health-related problems rated fairly often / very often in percent

Among the 14 OHIP-G14 items (as ranked in Fig. 1), the three most prevalent problems were:

1. “Life was less satisfying”.
2. “Everyday activities were more difficult”.
3. “Uncomfortable eating certain foods”.

Conversely, items related to difficulty pronouncing words and tension towards others were the least frequently impairing.

The overall mean OHIP-G14 severity score was 11.1.

### Bivariate analyses of factors associated with OHIP-G14 prevalence

In Table 3, we compared the proportions of respondents who answered “fairly often” or “very often” to at least one prevalent OHIP-G14 item with those who did not. Notably, OHIP-G14 prevalence exhibited a significant correlation with the level of care received in particular where, a higher level of care corresponded to a greater proportion of prevalent symptoms. Participants who did not have a dentist for regular visits had a higher proportion in the prevalence group. In this group, persons visited a dentist more frequently in the last 12 months. Utilisation of dental care since onset of HBC showed higher proportions in the prevalence group in both directions, using it more frequently or using it less frequently/ not at all. The subjective dental status was associated in both directions: lower proportions of oral problems were linked to a positive dental status, while higher proportions were prevalent among those with a negative dental status. Moreover, a lower prevalence of oral problems was observed in the group that brushed their teeth more frequently, as well as among older people who did not require support for oral and dental care, and in those participants with higher values in HRQoL. A higher prevalence of oral problems was seen in persons who reported subjective memory impairment.

**Table 3:** Prevalence of Oral Health-related Problems – at least one OHIP-G14 item categorised as present fairly often / very often

	Sum		Problems not prevalent		Problems prevalent		*p*-value
	**N**	**%**	**N**	**%**	**N**	**%**	
**Sex**							
Female	1,116	72.0	653	70.2	463	74.6	0.062
Male	435	28.0	277	29.8	158	25.4	
Sum	1,551	100.0	930	100.0	621	100.0	
**Age group**							
60–74 years	217	14.0	125	13.5	92	14.9	0.130
75–84 years	577	37.3	333	35.8	244	39.6	
>= 85 years	751	48.6	471	50.7	280	45.5	
Sum	1,545	100.0	929	100.0	616	100.0	
**Level of care**							
1	15	1.0	7	0.7	8	1.3	< 0.001
2	984	63.7	632	68.0	352	57.3	
3	409	26.5	220	23.7	189	30.8	
4	104	6.7	58	6.2	46	7.5	
5	32	2.1	13	1.4	19	3.1	
Sum	1,544	100.0	930	100.0	614	100.0	
**Education**							
Low	176	11.5	104	11.4	72	11.8	0.621
Medium	1,153	75.5	687	75.0	466	76.3	
High	198	13.0	125	13.6	73	11.9	
Sum	1,527	100.0	916	100.0	611	100.0	
**Living alone**							
Yes	758	51.0	459	50.9	299	51.0	0.976
No	729	49.0	442	49.1	287	49.0	
Sum	1,487	100.0	901	100.0	586	100.0	
**Have a preferred dentist**							
Yes	1,284	81.5	799	84.5	485	77.1	< 0.001
No	291	18.5	147	15.5	144	22.9	
Sum	1,575	100.0	946	100.0	629	100.0	
**Have a preferred GP**							
GP	1,397	91.4	844	91.8	553	90.7	0.420
Other	132	8.6	75	8.2	57	9.3	
Sum	1,529	100.0	919	100.0	610	100.0	
**Utilisation of dental care since HBC is needed**							
Unchanged	748	50.0	504	56.5	244	40.5	< 0.001
More frequently	112	7.5	47	5.3	65	10.8	
Less frequently / not at all	635	42.5	341	38.2	294	48.8	
Sum	1,495	100.0	892	100.0	603	100.0	
**Subjective dental status: mainly positive**							
Yes	1,113	70.7	784	82.9	329	52.3	< 0.001
No	462	29.3	162	17.1	300	47.7	
Sum	1,575	100.0	946	100.0	629	100.0	
**Subjective dental status: mainly negative**							
Yes	280	17.8	83	8.8	197	31.3	< 0.001
No	1,295	82.2	863	91.2	432	68.7	
Sum	1,575	100.0	946	100.0	629	100.0	
**Visited dentist in the past 12 months**							
Yes	1,093	78.7	661	79.3	432	78.0	0.569
No	295	21.3	173	20.7	122	22.0	
Sum	1,388	100.0	834	100.0	554	100.0	
**Frequency brushing teeth**							
Never	15	1.0	7	0.8	8	1.3	0.013
Irregular	76	5.0	39	4.3	37	6.1	
Once daily	306	20.3	167	18.5	139	23.1	
Twice daily	924	61.3	586	64.8	338	56.1	
More than twice daily	186	12.3	105	11.6	81	13.4	
Sum	1,507	100.0	904	100.0	603	100.0	
**Need support for oral care**							
Yes	405	26.3	176	19.0	229	37.5	< 0.001
No	1,133	73.7	751	81.0	382	62.5	
Sum	1,538	100.0	927	100.0	611	100.0	
**At least one person for support of oral care**							
No support	648	48.2	437	54.1	211	39.3	< 0.001
At least one person	697	51.8	371	45.9	326	60.7	
Sum	1,345	100.0	808	100.0	537	100.0	
**Subjective memory impairment**							
Yes	764	48.5	409	43.2	355	56.4	< 0.001
No	811	51.5	537	56.8	274	43.6	
Sum	1,575	100.0	946	100.0	629	100.0	
**Support to fill in questionnaire**							
Yes	730	46.3	429	45.3	301	47.9	0.329
No	845	53.7	517	54.7	328	52.1	
Sum	1,575	100.0	946	100.0	629	100.0	
**Number of dental visits in the past 12 months**							
	Mean	[95%-CI]	Mean	[95%-CI]	Mean	[95%-CI]	
	1.9	[1.8,2.1]	1.8	[1.6,1.9]	2.2	[1.9,2.4]	< 0.001
**HRQoL Eq. 5D**							
	Mean	[95%-CI]	Mean	[95%-CI]	Mean	[95%-CI]	
	0.275	[0.256,0.293]	0.353	[0.330,0.375]	0.161	[0.133,0.189]	< 0.001

Level of care 1 to 5 (1 = minor impairment of independence or abilities, 5 = most severe impairment of independence and/or abilities with special care requirements); OHIP-G14: Oral Health Impact Profile German Version 14 items; p-value: probability value for estimation of statistical significance; GP: General Practitioner; HBC: home-based care; HRQoL EQ5D: Health-Related Quality of Life EuroQoL 5 Dimensions; CI: confidence interval

### Multivariate associations with OHIP-G14 severity – CC group

Finally, we conducted a linear regression analysis to assess the severity of OHIP-G14 by integrating all variables and estimating the overall explained variance. The results of this regression, including β-coefficients and 95%-CI, are presented in Table 4. Several variables showed a significant negative association with OHIP-G14 severity, resulting in an interpretation of better OHRQoL: HRQoL, subjective dental health status was positive, and supported to complete the survey questionnaire.

**Table 4:** Multivariate regression for severity of oral health impacts (OHIP-G14)

	Beta-coefficient	Standard error	*p*-value	95%-Confidence interval below	95%-Confidence interval above
**Sex (ref = female)**	-0.7	0.7	0.344	-2.1	0.7
**Age**	0.05	0.04	0.176	-0.02	0.1
**Level of Care (ref = 1)**					
2	-4.0	2.8	0.149	-9.5	1.5
3	-3.5	2.8	0.216	-9.1	2.0
4	-4.7	3.0	0.121	-10.7	1.2
5	-7.5	3.6	0.040	-14.6	-0.3
**Education (ref = low)**					
Medium	-1.1	1.0	0.271	-3.0	0.8
High	-1.0	1.2	0.438	-3.4	1.5
**Living alone (ref = not living alone)**	1.6	0.6	0.017	0.3	2.8
**Have a regular dentist (ref = no)**	0.2	1.0	0.861	-1.7	2.1
**Have a regular GP (ref = no)**	-0.3	1.1	0.810	-2.4	1.9
**Utilisation of dental care since onset of HBC (ref = unchanged)**					
More frequently	1.6	1.2	0.190	-0.8	3.9
Less frequently / not at all	1.6	0.8	0.048	0.01	3.1
**Subjective dental status: mainly positive**	-8.2	1.0	< 0.001	-10.2	-6.2
**Subjective dental status: mainly negative**	4.7	1.2	< 0.001	2.8	7.1
**Visited dentist in the past 12 months (ref = no)**	1.7	1.0	0.077	-0.2	3.6
**Number of dental visits in the past 12 months**	0.8	0.2	< 0.001	0.5	1.2
**Frequency brushing teeth (ref = not at all)**					
Once daily	1.3	3.2	0.679	-4.9	7.6
Twice daily	1.6	3.2	0.603	-4.6	7.8
more than twice daily	2.4	3.3	0.466	-4.0	8.8
irregular	0.9	3.4	0.789	-5.8	7.7
**Need support for oral care (ref = no need)**	0.3	0.7	0.657	-1.1	1.7
**Number of persons for oral care > = 1 (ref = no person)**	3.6	0.9	< 0.001	1.9	5.4
**HRQoL Eq. 5D**	-8.3	0.9	< 0.001	-10.2	-6.4
**Subjective memory impairment**	2.6	0.6	< 0.001	1.4	3.8
**Support to fill in questionnaire**	-3.0	0.6	< 0.001	-4.3	-1.8

ref: category of reference for beta coefficient; Level of care 1 to 5 (1 = minor impairment of independence or abilities, 5 = most severe impairment of independence and/or abilities with special care requirements); GP: General Practitioner; HBC: home-based care; HRQoL EQ5D: Health-Related Quality of Life EuroQoL 5 Dimensions

The following variables had a significant positive association with OHIP-G14 severity, resulting in an estimation of poorer OHRQoL: participants living alone, having a negative subjective dental health status, reporting a higher number of dental visits in the past 12 months, and postulating that at least one person is available to help with oral care. Participants who saw a dentist less frequently or not at all since the onset of HBC compared with the group who visited a dental practice with no change showed poorer OHRQoL. The same applies to participants who admitted that their cognitive abilities were declining.

In the regression model, no associations were observed between OHRQoL and variables such as sex, age, education, preference for a GP as the initial point of medical contact, need for oral care support, frequency of brushing teeth, having a preferred dentist, or dental visits within the past 12 months.

The regression model demonstrated a finding of a linear decrease in oral health problems as care levels increased. Statistical significance was observed specifically, when comparing care level 1 to care level 5.

Overall, this regression model accounted for an adjusted 41.7% of the variance in the model. For cross-validation purposes, we also developed a logistic model for the OHIP-G14-prevalence and a linear regression model for the OHIP-G14-extent (not presented here). Both analyses yielded similar results regarding the factors associated with OHRQoL in the CC group. However, neither model performed as good as the preferred model described in this paper in terms of explaining variance. Although, the logistic regression with OHIP-G14 prevalence explained 22.3% of the variance, the multivariate model with OHIP-G14 extent accounted for an adjusted explanatory variance of 33.6%. Ultimately, the model with the least loss of information (severity) was most effective in explaining variation in its components.

## Discussion

This survey focused on older people receiving HBC in Hamburg, Germany. The study employed a dPROM (OHIP-G14), to investigate the OHRQoL. However, five factors were identified that increased the severity score of OHRQoL, indicating greater impairment: a mainly negative subjective dental health status, need for support in OHC, presence of a designated support person, living alone, and subjective memory impairment. But, three factors were also associated with lower OHRQoL severity scores, indicating less impairment: a mainly positive subjective dental health status, a better HRQoL measured with the EQ5D, and assistance in completing the questionnaire. Henni and colleagues [18] highlighted the scarcity of evidence on the OHRQoL of older people in HBC. This study contributes to filling that knowledge gap by affirming that this group, particularly those perceiving their dental health negatively and require assistance in OHC is at risk of poor OHRQoL, a phenomenon well-documented among nursing home residents [16, 20]. Germany shows a discrepancy in oral health between inpatient and outpatient settings: older people requiring outpatient care tend to have poorer oral health than those living in nursing homes. Collaboration agreements between dentists and nursing homes have been shown to enhance the utilisation of dental services, care tailored to specific needs [40].

The study results also indicated that older people living alone were at risk for poor oral health, which aligns with the findings of Lindmark et al. [41]. However, Jensen et al. [42] did not observe an increased risk for older people living alone. These conflicting results imply that living alone may not be a reliable indicator of loneliness. Furthermore, the study revealed a correlation between cognitive impairment and self-reported poor oral health, which has been consistent with previous research [43, 44].

The current study found a positive association between OHRQoL and perceived good dental health.

Part of the reason for this is the overlap between inquiries regarding subjective dental health status and those concerning OHRQoL. Additionally, in consistence with previous research, a good overall HRQoL was linked to improved OHRQoL [45, 46]. In our sample, particularly 12.0% reported four or more oral health-related impairments fairly often or very often in the four weeks preceding their response to the OHIP-G14 measurement. Furthermore, the reported overall severity score was considerably higher compared to other German studies within the same age group [47, 48].

In our study involving older people requiring HBC, we identified several risk factors associated with poor OHRQoL. Although the impact of these factors was relatively minor, it is crucial to recognise that older people living alone with HBC may require specific attention from both professional and non-professional caregivers regarding oral and dental care. This argument gains further support from our findings: participants who acknowledged needing support with oral and dental care were at risk for poor OHRQoL, as were those who self-evaluated their dental status negatively. Thus, this pattern suggests that older people in HBC often recognise their dental situation as poor or negative, even among the subgroup reporting subjective memory impairment. This trend persisted strikingly among older people who had visited a dentist more frequently in the past 12 months. However, it is essential to recognise that the OHIP-G14, as a measurement tool for OHRQoL, was not specifically designed to differentiate between the objective care situations (such as frequent dental visits) and the subjective need for support in oral and dental care. Consequently, its interpretation must be confined to the specific investigative context in which it is employed. Interestingly, participant’s subjective dental status revealed a phenomenon that could serve as cross-validation for the OHRQoL assessment. Specially for older people who negatively evaluated their dental and oral conditions based on the four status items (teeth, mucosa/tongue/gums, dentures, and dental care) and also reported significantly poorer OHRQoL. Conversely, a positive self-evaluation of one’s own dental status was associated with a clearly positive QHRQoL.

On the one hand, the lack of surprise stemmed from the fact that the operationalisation of both constructs, dental status and OHRQoL, encompassed comparable domains of concerns that are described [49]. On the other hand, the magnitude and clarity of the observed disparity, reaching a non-standardised mean difference of 13 OHIP points between the delineated subgroups, was indeed unexpected. This suggests that our sample of older people in HBC demonstrated adeptness in evaluating their oral health status, irrespective of their capacity or inclination to seek professional dental care.

This observation is supported by a discovery from the bivariate analysis: older people indicating a higher frequency of oral health issues on the OHIP-G14 questionnaire, specifically categorised as occurring fairly often or very often, have utilised more dental services since onset of HBC. Nevertheless, those who have utilised fewer dental services since onset of HBC also exhibit significantly more issues on the OHIP-G14 questionnaire, occurring fairly often or very often. While initially appearing contradictory, closer scrutiny elucidates that older people who have seen a dentist more frequently since onset of HBC were more likely to have established a regular dental care regimen, whereas those who utilised dental services less frequently or not at all despite experiencing oral health complaints were more likely to report lacking a regular dentist.

Despite the finding that the general frailty is proposed as a significant contributor to diminished OHRQoL [50], our regression model suggested that increased level of care is statistically associated with less impaired OHRQoL. This finding appears to be a statistical artefact due to missing values of older people with a higher level of care.

Qualitative research by Niesten et al. [51, 52] provided additional insights into the factors influencing poor OHRQoL, including inadequate financial incentives for collaborative practices, fragmentation within the health care system, insufficient integration of OHC into care procedures, instruments and guidelines.

Moreover, older people reporting lower prevalence, extent, and severity of oral health impacts demonstrated a better HRQoL. This findings aligns with existing literature, as both quality-of life-measurements exhibit consistent associations: better OHRQoL corresponds to better HRQoL, and vice versa [53–55].

This study reveals the high prevalence of oral health impacts among a large proportion of older people receiving HBC. The findings support assertion that this population is susceptible to compromised oral health, even though over 70.0% of the sample had visited a dentist in the past 12 months. Negative self-assessment of dental status and subjective memory impairment were associated with increased extent and severity of oral health impacts.

### Strengths and limitations

As indicated by literature reviews, investigations of OHRQoL, dental, and oral care of older people requiring HBC have been limited thus far. Our study contributes to this body of knowledge.

A notable strength of our survey was the inclusion of a large sample of older people receiving HBC, yielding a commendable response rate, which was considered unusual by the responsible statutory health insurance company, DAK-Gesundheit. Additionally, our study benefited from the comprehensive survey of all insured older people listed in the DAK-Gesundheit database at the time, adhering to strict inclusion criteria.

Furthermore, the distribution of age, sex and, to some extent, care levels among respondents was another strength. Comparisons with the total sample from DAK-Gesundheit records revealed minor differences: 26.2% of the total sample were male (compared to 28.0% of responders), the mean age was 82.7 years (compared to 83.2 years for responders), and the proportion of older people with care level 2, the largest group, was 58.7% (compared to 63.6% of responders). Notably, the distribution of care levels slightly favoured those with lower care needs, a trend consistent across higher care levels, likely influenced by the heightened physical impairment observed particularly in levels 4 and 5.

While the latter is certainly a limitation, several others exist. Firstly, our sample comprised older people insured solely with DAK-Gesundheit, excluding participants from other statutory health insurance companies. This exclusivity may introduce bias, especially considering that nearly half of participants reported subjective memory impairment. Given the substantial variability in socio-economic, socio-demographic, and morbidity characteristics among older people insured by different statutory health insurance companies in Germany, caution must be exercised when generalising findings about older people receiving HBC.

Moreover, the study does not provide information about the actual oral health situation. This aspect is addressed in subproject 3, another part of the InSEMaP study aimed at answering this specific question.

### Implication for practice and future research directions

Tomar & Cohen [56] delineated an ideal OHC system that integrates seamlessly with the broader health care framework, emphasising health promotion and disease prevention. The envisioned system aspires to be “evidence-based, effective, cost-effective, sustainable, equitable, universal, comprehensive, ethical […]” among other qualities. Given that older people receiving HBC constitute a vulnerable population concerning oral and dental health, significant challenges arise due to limited time allocated for daily home care, leaving insufficient room for oral care [57].

As the barriers to accessing OHC escalate among older people receiving HBC, there may be a call for authorities to intervene. In Germany, the Medical Service holds the responsibility for assessing care needs and assigning care levels [25]. This regulatory body, entrusted with routinely evaluating the care needs of older people, could integrate a screening mechanism for OHC within their evaluation process, ensuring that the oral health needs of older people receiving HBC are not overlooked. Further research within the InSEMaP study framework will delve into health insurance claims data to explore reasons for discontinuation of oral health care, while also investigating the association between systemic morbidity and OHC (subproject 2). Additionally, a forthcoming path of research will involve assessing oral care and dental health through clinical examinations conducted in participants’ home as a part of subproject 3.

## Conclusion

The results highlight the risk for poor oral health among older people in HBC. We conclude that there is an urgent need to prioritise oral health, especially as poor oral health can further compromise the systemic wellbeing of these already care dependent older people.

### Electronic supplementary material

Below is the link to the electronic supplementary material.

Supplementary Material 1

Supplementary Material 2

## Data Availability

The datasets generated and/or analysed during the current study are not publicly available due to compliance with German social law. Data can be requested via the corresponding author. As different institutions are involved in the InSEMaP study group (consortium), there will be a process implemented to evaluate research questions and check methodology of the data request for soundness.

## Declarations

### Registration

The study was prospectively included in the German Clinical Trials Register (DRKS) on 04/01/2022: DRKS00027020. https://drks.de/search/de/trial/DRKS00027020.

## Abbreviations

DAK-Gesundheit: Deutsche Angestellten Krankenkasse – Gesundheit
DRKS: German Clinical Trials Register
EQ5D: European Quality of Life five Dimensions
GP: General Practitioner
HBC: home-based Care
HRQoL: Health-Related Quality of Life
InSEMaP: Interaction of Systemic Morbidity and Oral Health in Ambulatory Patients in Need of Home Care
OHC: Oral Health Care
OHIP-G14: German short version of Oral Health Impact Profile
OHRQoL: Oral Health-Related Quality of Life
