# Supplementary material for: Assessing oral health-related quality of life among older people in home-based care - survey results of the InSEMaP study in Germany
Source: BMC Oral Health. 2024 Jun 26;24:734. doi: 10.1186/s12903-024-04500-6 (PMC11209957; doi:10.1186/s12903-024-04500-6)
Supplement: Supplementary file 1 — Supplementary Material 1 [file 12903_2024_4500_MOESM1_ESM.pdf]

Fragebogen zu Ihrer mundgesundheitlichen Versorgung

Bitte beantworten Sie nach Möglichkeit alle Fragen. Wählen Sie nur eine Antwortmöglichkeit aus, es sei denn in der Frage wird darauf hingewiesen, dass mehrere Antworten ausgewählt werden können.

Falls Sie sich beim Ausfüllen unterstützen lassen, kreuzen Sie bitte dieses Kästchen an: ☐

Angaben zu meinem Unterstützungsbedarf

1. Mein aktueller Pflegegrad ist:
☐ keiner ☐ 1 ☐ 2 ☐ 3 ☐ 4 ☐ 5

2. Mein aktueller Pflegegrad besteht seit (Jahresangabe):

3. Erstmals wurde mir ein Pflegegrad / eine Pflegestufe bescheinigt im Jahr:

4. In meinem praktischen Alltag werde ich unterstützt von:
☐ niemandem ☐ einer Person ☐ zwei Personen
☐ drei Personen ☐ mehr als drei Personen

5. Diese Personen sind: *(hier können mehrere Antworten ausgewählt werden)*
☐ Ehe- oder Lebenspartner ☐ Angehörige ☐ Freunde / Nachbarn
☐ ambulanter Pflegedienst ☐ sonstige Personen ☐ Ich habe keine Unterstützungsperson.

6. Ich lebe …
☐ allein.
☐ mit mindestens einer Person, die mich unterstützt.
☐ Beide vorherigen Antworten treffen nicht zu.

7. Ich benötige Unterstützung bei der Mund- und Zahnpflege.
☐ keine ☐ teilweise ☐ vollständig

8. Ich erhalte bei Bedarf Unterstützung bei der Mund- und Zahnpflege durch: *(hier können mehrere Antworten ausgewählt werden)*
☐ Ehe- oder Lebenspartner ☐ Angehörige ☐ Freunde / Nachbarn
☐ ambulanter Pflegedienst ☐ sonstige Personen ☐ Ich habe keinen Bedarf.

Angaben zu meiner zahnärztlichen Versorgung

9. In den vergangenen 12 Monaten hatte ich insgesamt ____ (Anzahl) zahnärztliche Untersuchungen.

10. Ich wurde bereits zu Hause zahnärztlich versorgt.
☐ ja ☐ nein

11. Ich habe einen festen Zahnarzt.

☐ ja ☐ nein *(weiter mit **12.**)* ☐ weiß ich nicht *(weiter mit **12.**)*

Dieser Zahnarzt führt im Bedarfsfall Hausbesuche durch.

☐ ja ☐ nein ☐ weiß ich nicht

Dieser Zahnarzt ist über meinen allgemeinen Gesundheits- und Pflegezustand informiert.

☐ ja ☐ nein ☐ weiß ich nicht ☐ teils teils

Dieser Zahnarzt tauscht sich mit anderen Ärzten über meine Mund- und Zahngesundheit aus.

☐ ja ☐ nein ☐ weiß ich nicht

12. Seit Eintritt meiner Pflegebedürftigkeit nehme ich zahnärztliche Untersuchungen in Anspruch:

☐ häufiger (weiter mit **13.**) ☐ unverändert (weiter mit **13.**) ☐ seltener ☐ gar nicht mehr

Ich war seit Eintritt meiner Pflegebedürftigkeit seltener bzw. gar nicht mehr beim Zahnarzt, weil … *(hier können mehrere Antworten ausgewählt werden)*

- ☐ ich keinen Bedarf hatte.
- ☐ anderes für mich wichtiger ist.
- ☐ die Terminorganisation schwierig ist.
- ☐ ich zu große Bewegungsprobleme habe.
- ☐ die Zahnarztpraxis nicht barrierefrei ist.
- ☐ mir die Unterstützung fehlt, um in die Zahnarztpraxis zu gelangen.
- ☐ die Kosten der Behandlung für mich zu hoch sein könnten.
- ☐ ich Angst vor der Behandlung habe.
- ☐ weitere Gründe:

Ich weiß, dass ich zahnärztliche Behandlungen benötige, nehme sie aber nicht in Anspruch, weil … *(hier können mehrere Antworten ausgewählt werden)*

- ☐ Aussage trifft auf mich nicht zu.
- ☐ anderes für mich wichtiger ist.
- ☐ die Terminorganisation schwierig ist.
- ☐ ich zu große Bewegungsprobleme habe.
- ☐ die Zahnarztpraxis nicht barrierefrei ist.
- ☐ mir die Unterstützung fehlt, um in die Zahnarztpraxis zu gelangen.
- ☐ die Kosten der Behandlung für mich zu hoch sein könnten.
- ☐ ich Angst vor der Behandlung habe.
- ☐ weitere Gründe:

Angaben zu meiner Mundgesundheit

16. Ich habe … *(hier können mehrere Antworten ausgewählt werden)*

- ☐ eigene Zähne.
- ☐ Implantate.
- ☐ einen festsitzenden Zahnersatz.
- ☐ eine herausnehmbare Teilprothese.
- ☐ eine Vollprothese.
- ☐ keine eigenen Zähne mehr.

17. Meine Mund- und Zahnpflege wird durchgeführt:

- ☐ gar nicht
- ☐ einmal täglich
- ☐ zweimal täglich
- ☐ mehr als zweimal täglich
- ☐ unregelmäßig

**18. Ich schätze ein:**	**Sehr gut**	**Eher gut**	**Eher schlecht**	**Sehr schlecht**		**Trifft nicht zu**
Zustand meiner Zähne	☐	☐	☐	☐		☐
Zustand von Schleimhaut/ Zunge/ Zahnfleisch	☐	☐	☐	☐		☐
Zustand meines Zahnersatzes	☐	☐	☐	☐		☐
meine zahnärztliche Versorgung	☐	☐	☐	☐		☐

Angaben zu meiner ärztlichen Versorgung

19. Meine erste ärztliche Ansprechperson, wenn ich ein Gesundheitsproblem habe, ist:

- ☐ mein Hausarzt
- ☐ ein Arzt einer anderen Fachrichtung
  Bitte hier die Fachrichtung eintragen: ______
- ☐ Ich habe keine ärztliche Ansprechperson.

20. Diese ärztliche Ansprechperson führt im Bedarfsfall auch <u>Hausbesuche</u> durch.

☐ ja ☐ nein ☐ weiß ich nicht

21. In den vergangenen 12 Monaten hatte ich insgesamt Kontakt zu dieser ärztlichen Ansprechperson: *(alle Besuche mit direktem Arztkontakt sowie Hausbesuche, nicht nur Rezepte abholen)* ______ *mal*

22. Diese ärztliche Ansprechperson ist über meine Mund- und Zahngesundheit informiert.

☐ ja ☐ nein ☐ weiß ich nicht

23. Diese ärztliche Ansprechperson würde mir bei Bedarf eine zahnärztliche Behandlung empfehlen.

☐ ja ☐ nein ☐ weiß ich nicht

24. Diese ärztliche Ansprechperson tauscht sich mit meinem Zahnarzt aus.

☐ ja ☐ nein ☐ weiß ich nicht

Angaben zu meiner heutigen Gesundheit
Bitte geben Sie an, welche Aussagen Ihren **heutigen Gesundheitszustand** am besten beschreiben, indem Sie die entsprechende Aussage einer Gruppe ankreuzen.

25. Beweglichkeit / Mobilität
- ☐ Ich habe keine Probleme herumzugehen.
- ☐ Ich habe leichte Probleme herumzugehen.
- ☐ Ich habe mäßige Probleme herumzugehen.
- ☐ Ich habe große Probleme herumzugehen.
- ☐ Ich bin nicht in der Lage herumzugehen.

26. Für sich selbst sorgen
- ☐ Ich habe keine Probleme, mich selbst zu waschen oder anzuziehen.
- ☐ Ich habe leichte Probleme, mich selbst zu waschen oder anzuziehen.
- ☐ Ich habe mäßige Probleme, mich selbst zu waschen oder anzuziehen.
- ☐ Ich habe große Probleme, mich selbst zu waschen oder anzuziehen.
- ☐ Ich bin nicht in der Lage, mich selbst zu waschen oder anzuziehen.

27. Alltägliche Tätigkeiten (z.B. Hausarbeit, Familien- oder Freizeitaktivitäten)
- ☐ Ich habe keine Probleme, meinen alltäglichen Aktivitäten nachzugehen.
- ☐ Ich habe leichte Probleme, meinen alltäglichen Aktivitäten nachzugehen.
- ☐ Ich habe mäßige Probleme, meinen alltäglichen Aktivitäten nachzugehen.
- ☐ Ich habe große Probleme, meinen alltäglichen Aktivitäten nachzugehen.
- ☐ Ich bin nicht in der Lage, meinen alltäglichen Aktivitäten nachzugehen.

28. Schmerzen / körperliche Beschwerden
- ☐ Ich habe keine Schmerzen oder Beschwerden.
- ☐ Ich habe leichte Schmerzen oder Beschwerden.
- ☐ Ich habe mäßige Schmerzen oder Beschwerden.
- ☐ Ich habe starke Schmerzen oder Beschwerden.
- ☐ Ich habe extreme Schmerzen oder Beschwerden.

29. Angst / Niedergeschlagenheit
- ☐ Ich bin nicht ängstlich oder deprimiert.
- ☐ Ich bin ein wenig ängstlich oder deprimiert.
- ☐ Ich bin mäßig ängstlich oder deprimiert.
- ☐ Ich bin sehr ängstlich oder deprimiert.
- ☐ Ich bin extrem ängstlich oder deprimiert.

30. Wir wollen herausfinden, wie gut oder schlecht Ihre Gesundheit **HEUTE** ist. Diese Skala ist mit Zahlen von 0 bis 100 versehen. Bitte kreuzen Sie den Punkt auf der Skala an, der Ihre Gesundheit **HEUTE** am besten beschreibt.

0 10 20 30 40 50 60 70 80 90 100

0 = Schlechteste Gesundheit, die Sie sich vorstellen können

100 = Beste Gesundheit, die Sie sich vorstellen können

31. Ich habe das Gefühl, mein Gedächtnis wird schlechter.

☐ ja ☐ nein

Wenn ja, das macht mir Sorgen.

☐ ja ☐ nein

Angaben zu meiner Person

32. Mein Alter: ____ Jahre

33. Ich bin: ☐ männlich ☐ weiblich ☐ divers

34. Mein <u>höchster</u> beruflicher Bildungsabschluss ist:

- ☐ Ich habe keinen Abschluss
- ☐ Hauptschulabschluss ohne berufliche Ausbildung
- ☐ Hauptschulabschluss mit beruflicher Ausbildung
- ☐ Mittlere Reife ohne berufliche Ausbildung
- ☐ Mittlere Reife mit beruflicher Ausbildung
- ☐ Fachhochschulreife / Abitur ohne berufliche Ausbildung
- ☐ Fachhochschulreife / Abitur mit beruflicher Ausbildung
- ☐ Fachhochschulabschluss
- ☐ Universitätsabschluss

35. Ich komme mit meinem Geld gut über die Runden.

☐ ja ☐ es geht so ☐ nein, schlecht

36. Beim Ausfüllen des Fragebogens wurde ich unterstützt durch:

☐ Ehe- oder Lebenspartner ☐ Angehörige ☐ Freunde / Nachbarn

☐ ambulanter Pflegedienst ☐ sonstige Personen ☐ Ich hatte keine Unterstützung.

Legen Sie den ausgefüllten Fragebogen bitte in den beigelegten Umschlag und senden ihn an uns zurück.

Ihre Mithilfe ist ein wichtiger Beitrag!
Wir danken Ihnen für Ihre Teilnahme.
